# The Polyploid Series of the *Achillea millefolium* Aggregate in the Iberian Peninsula Investigated Using Microsatellites

**DOI:** 10.1371/journal.pone.0129861

**Published:** 2015-06-19

**Authors:** Sara López-Vinyallonga, Ignasi Soriano, Alfonso Susanna, Josep Maria Montserra, Cristina Roquet, Núria Garcia-Jacas

**Affiliations:** 1 Botanic Institute of Barcelona (IBB-CSIC-ICUB), Pg. del Migdia, Barcelona, Spain; 2 Department of Plant Biology, University of Barcelona, Barcelona, Spain; 3 Barcelona Botanical Garden (Consortium of the Museum of Natural History of Barcelona), Barcelona, Spain; 4 Laboratoire d'Ecologie Alpine, CNRS UMR 5553, Université Grenoble Alpes, Grenoble, France; National Cheng-Kung University, TAIWAN

## Abstract

The *Achillea millefolium* aggregate is one of the most diverse polyploid complexes of the Northern hemisphere and has its western Eurasian boundary in the Iberian Peninsula. Four ploidy levels have been detected in *A*. *millefolium*, three of which have already been found in Iberia (diploid, hexaploid and octoploid), and a fourth (tetraploid) reported during the preparation of this paper. We collected a sample from 26 Iberian populations comprising all ploidy levels, and we used microsatellite markers analyzed as dominant in view of the high ploidy levels. Our goals were to quantify the genetic diversity of *A*. *millefolium* in the Iberian Peninsula, to elucidate its genetic structure, to investigate the differences in ploidy levels, and to analyse the dispersal of the species. The lack of spatial genetic structure recovered is linked to both high levels of gene flow between populations and to the fact that most genetic variability occurs within populations. This in turn suggests the existence of a huge panmictic yarrow population in the Iberian Peninsula. This is consistent with the assumption that recent colonization and rapid expansion occurred throughout this area. Likewise, the low levels of genetic variability recovered suggest that bottlenecks and/or founder events may have been involved in this process, and clonal reproduction may have played an important role in maintaining this genetic impoverishment. Indeed, the ecological and phenologic uniformity present in the *A*. *millefolium* agg. in Iberia compared to Eurasia and North America may be responsible for the low number of representatives of this complex of species present in the Iberian Peninsula. The low levels of genetic differentiation between ploidy levels recovered in our work suggest the absence of barriers between them.

## Introduction

*Achillea millefolium* agg. is one of the most diverse polyploid complexes of the Northern hemisphere in terms of morphological, genetic and ecological features [1–4]. This group includes *Achillea millefolium*, together with a set of Eurasian and North American related lineages (ca. 24 species; [2]), some of them naturalized in temperate and cold areas on other continents. In Eurasia, the group has its southwestern boundary in the Iberian Peninsula, where it is widely distributed throughout the extra-Mediterranean areas of the northern half, reaching some southern mountain ranges. Yarrows are plants of economic importance, widely used in agriculture and horticulture, as well as in pharmacology and folk medicine due to their numerous therapeutic properties. For example, they are considered to contain antihypertensive, anti-inflammatory, antimalarial and antimicrobial compounds, and have a long list of other uses beside [5–11].

According to [4], the complexity of the aforementioned group is the result of multiple processes of hybridization, polyploidization and evolution linked with different types of habitats. Furthermore, the naturalization of alien strains, introduced either intentionally or accidentally, contributed to this. The existence of different auto- and allopolyploids representing four ploidy levels (2*x*, 4*x*, 6*x* and 8*x*) is widely documented [4], and analysis of genetic diversity carried out using AFLP markers revealed substantial polymorphism, significantly higher in polyploid than in diploid strains [3]. All our knowledge of the polyploidy and genetics of this species is based on taxa and populations originating from central Eurasia, where the main diversity of the group is concentrated, and, to date, genetic studies on the Iberian populations have not been undertaken. Based on their own data from the northern slopes of the Pyrenees and on few previously published chromosome counts from Portugal [13] and France [14], [3,12] suggest the existence of three entities on the Iberian Peninsula corresponding to at least three ploidy levels whose distribution is poorly known: 2*x* (often considered a different species, *A*. *ceretanica*, endemic to the eastern Pyrenees), 6*x* (*A*. *millefolium s*. *str*.) and 8*x* (sometimes considered another different species, *A*. *monticola*). The hexaploid and the octoploid cytotypes are difficult to distinguish based on morphological criteria (I. Soriano in *Flora Iberica*, unpublished report), and genetic data show no clear differences [1], [12]. The presence of the three karyologic entities in Iberia has been confirmed by indirect methods (flow cytometry and stomatal measurements of herbarium material) together with chromosome counts (I. Soriano, University of Barcelona, unpublished data; see a breakthrough in [15]). The distribution of the three cytotypes is still unclear, although the 8*x* seem to be much more common than the 6*x* populations. The tetraploid cytotype had not been reported from the Iberian Peninsula, but we have recorded its presence there (albeit very scarce) during the research carried out for this paper (A. Susanna et al., Botanic Institute of Barcelona, unpublished data). Hexaploid strains are considered weeds and display a sub-cosmopolitan distribution [3,16]. In the Iberian Peninsula, *A*. *millefolium* agg. does not display the great ecological diversity present in Eurasia and North America. Instead, their populations tend to occupy ruderal and nitrified environments and disturbed pastures.

The lack of genetic studies on Iberian yarrows motivated us to investigate this species aggregate at population level using SSR markers (microsatellites). Microsatellites are widely used to investigate genetic variation, structure and dynamics both at population and species level (e. g., [17,18]). The main advantages of microsatellites are: i) high polymorphism derived from a high mutation rate; ii) co-dominant Mendelian inheritance; and iii) multi-allelic nature [19]. In polyploid organisms, the number of alleles for each locus is not known in partial heterozygotes. In these cases, the number of microsatellite DNA alleles displayed is less than the possible maximum number for the ploidy level of a given individual at a given locus [20,21]. Therefore, it is not possible to calculate some of the usual parameters of genetic diversity [22–25]. For this reason, despite polyploidy being common in plants [26–30], few studies on population genetics using microsatellites focus on these organisms [20]. However, different approaches have been proposed to overcome these problems [20–23,31–34] and microsatellites are an adequate tool for this purpose.

The aims of the present work are: (i) to quantify the genetic diversity of *A*. *millefolium* in the Iberian Peninsula; (ii) to elucidate its genetic structure and gene flow; (iii) to investigate the putative genetic differentiation between ploidy levels; and (iv) to evaluate alternative dispersal methods for this species within the reported area.

## Materials and Methods

### Plant material

**Fig 1:**
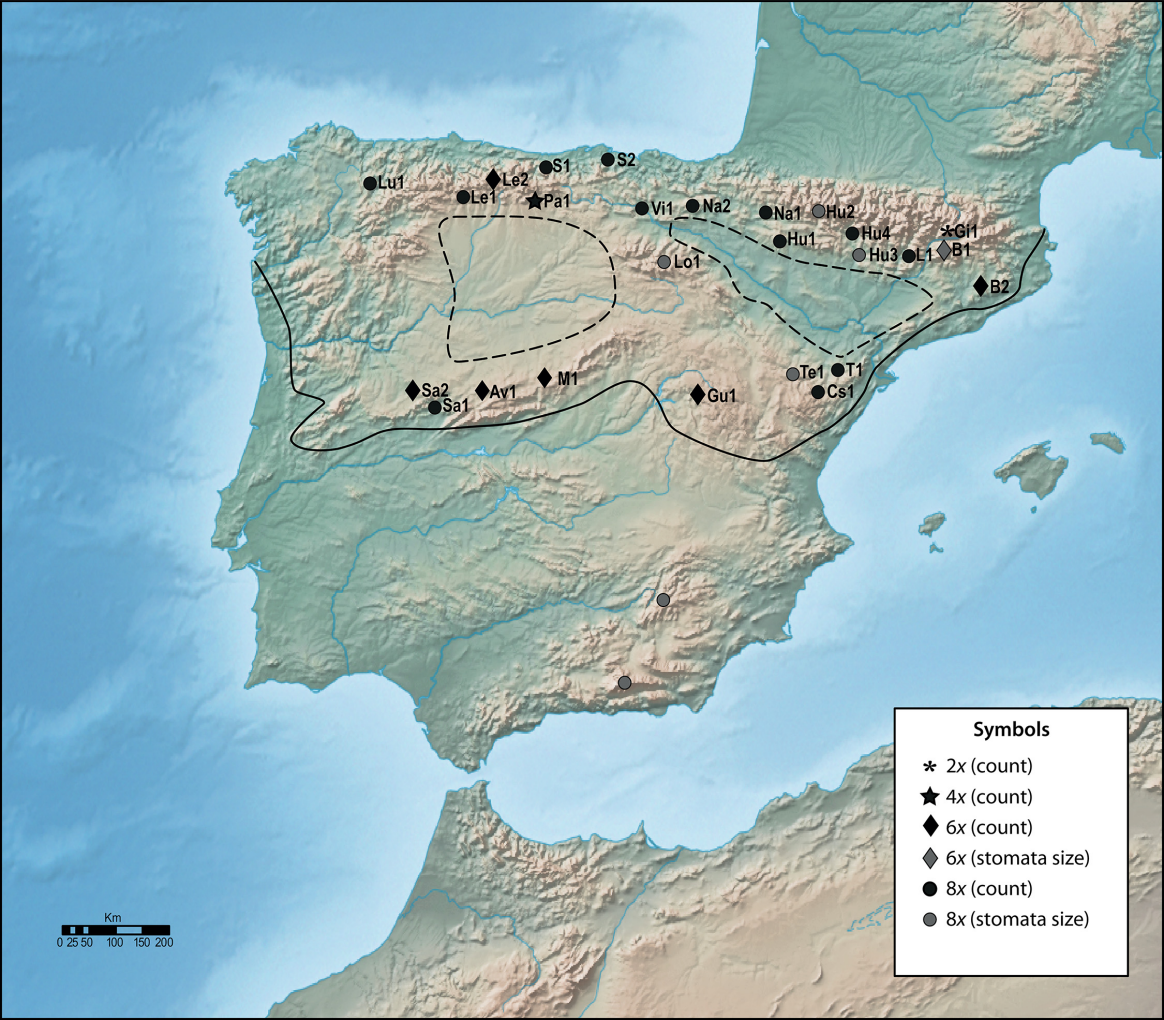
Locations of sampling with indication of ploidy level. A solid line indicates the southern limit of the area of *A*. *millefolium*. Within this area, dotted lines delimit the areas where the species is absent. The two points in South Iberia represent the most probable localities where the species currently grows (see “Plant material” for details). Base map from Natural Earth (http://www.naturalearthdata.com/).

Our sampling covered the whole area of *A*. *millefolium* in the Iberian Peninsula (Fig 1), with only one exception, namely, that despite intense searching, we were not able to include the populations reported from southern Iberia. Some of these citations are perhaps best considered cases of naturalization, involving individuals that have escaped from cultivation [Gabriel Blanca, University of Granada (Spain), pers. comm.]. A total of 26 populations, each containing 12–16 individuals, were sampled for the study (Table 1). Species of the *Achillea millefolium* aggregate are not endangered or protected in Spain and the EU, and our sampling was not carried out in any protected area. Thereafter, no specific permissions were required for our collections.

**Table 1:** Populations of *Achillea millefolium* investigated in the present study. K: chromosome count; S: stomatal size. Altitudes are expressed in meters above sea level.

Code	Location	Coordinates and Altitude	Habitat	Ploidy	Method
Av1	ÁVILA: between Poveda and Pradosegar	40° 33.6480’N 5° 5.5512’W; 1,171 m	Meadows and dry siliceous grasslands	6x	K + S
B1	BARCELONA: Pobla de Lillet, heliport	42° 14.6460’N 1° 57.8028’E, 887 m	Meso-xerophilous calcareous pastures and edges of paths.	6x	S
B2	BARCELONA: Montseny massif	41° 48.3552’N 2° 21.2484’E; 1,326 m	Mesophilous siliceous pastures and thickets.	6x	K + S
Cs1	CASTELLÓ: road CV15 Vilafranca-coll d'Ares	40° 27.2088’N 0° 8.6106W; 1,145 m	Damp patches on calcareous fringes	8x	K + S
Gi1	GIRONA: Alp, La Molina	42° 20.0496’N 1° 55.9392’E; 1,647 m	Mesophilous siliceous pastures and ski slopes.	2*x*	K + S
Gu1	GUADALAJARA: Orea	40° 33.,8544N 1° 44.5692W; 1,466 m	Field margins and slopes, near a stream.	6*x*	K + S
Hu1	HUESCA: Loarre Castle	42° 19.3258’N 0° 37.0019’W; 1,040 m	Waste places and roadsides on calcareous soil	8*x*	K + S
Hu2	HUESCA: Valle de Tena, Barranco del Petruso	42° 47.7942’N 0° 24.0918’W, 1,700 m	Waste places and roadsides on calcareous soil	8*x*	S
Hu3	HUESCA: Escales reservoir	42° 20.3377’N 0° 43.6887E; 816 m	Meso-xerophilous calcareous pastures.	6*x*	S
Hu4	HUESCA: Plan	42° 34.8500’N 0° 20.5110 E; 1,085 m	Oak fringes and edges of paths.	8*x*	K
L1	LLEIDA: Vall de la Vansa, Clot de les Fonts	42° 14.3322’N 1° 26.0190E; 870 m	Waste places on calcareous soil	8*x*	K
Le1	LEÓN: Pola de Gordón	42° 51.0504’N 5° 39.8982’W; 1,107 m	Clearings, slopes and meadows, near a river.	8*x*	K
Le2	LEÓN: Mampodre massif	43° 2.2848’ 5° 12,0774’W; 1,810 m	Mesophilous calcareous pastures.	6*x*	K
Lo1	LA RIOJA: Viniegra de Abajo, Collado Ocejo	42° 12.5798’W 2° 54.0270’W; 1,788 m	Mesophilous siliceous pastures.	8*x*	S
Lu1	LUGO: Bullán, Quinta de Cancelada, Becerreá	42° 58.0902’N 7° 5.0967W; 725 m	Mesophilous siliceous pastures	8*x*	K
M1	MADRID: Sierra de Guadarrama, Fuente de los Geólogos	40° 46.5521’N 4° 0.3374’W; 1,743 m	Scots pine fringes and slopes, near a pic-nic area.	6*x*	K + S
Na1	NAVARRA: Bigüezal	42° 41.0916N 1° 8.3124’W; 880 m	Meadows on calcareous soil.	8*x*	K + S
Na2	NAVARRA: Sierra de Urbasa, south from Alsasua	42° 52.3212N 2° 10.9782W; 683 m	Oak and beechwood fringes and edges of paths.	8*x*	K + S
Pa1	PALENCIA: Cervera de Pisuerga	42° 52.0194N 4° 30.1638W; 1029 m	Clearings with dry meadows, near the road	4*x*	K
S1	CANTABRIA: road CA-182 to Cabuérniga	43° 15.1908’N 4° 23.0844W; 155 m	Meadows, slopes and roadsides.	8*x*	K + S
S2	CANTABRIA: Laredo	43° 24.8262’N 3° 24.2904’W; 26 m	Meadows and edges of paths, near the coastline.	8*x*	K + S
Sa1	SALAMANCA: between Candelario and La Garganta	40° 20.3934’N 5° 46.0698’W; 1,220 m	Oakwood fringes and edges of paths.	8*x*	K
Sa2	SALAMANCA: La Alberca	40° 29.3124’N 6° 6.9036’W; 1,085 m	Scots pine fringes and edges of paths.	6*x*	K + S
T1	TARRAGONA: Port de Tortosa, Cova de les Avellanes	40° 47.3028’N 0° 18.3984’E; 990 m	Oakwood fringes and edges of paths.	8*x*	K
Te1	TERUEL: road N-420 between Valdeconejos and Utrillas	40° 46.616’N 0° 49.863W; 1,258 m	Waste places on calcareous soil.	8*x*	S
Vi1	ÁLAVA: between Paul and Salinas de Añana	42° 47.8578N 2° 58.1724W; 676 m	Slopes and roadsides.	8*x*	K + S

### Chromosome counts

Chromosome preparations were obtained using root meristems gathered from germinating seeds or from wild-collected plants cultivated at the Botanic Garden of Barcelona. Root tips were first pretreated with 0.002 M 8-hydroxyquinoline at 4°C for 8 h or in 0.02% colchicine at room temperature for 3 h, then the material was fixed with Carnoy at low temperatures for 24 h. Root tips were then hydrolysed with 5N HCl at room temperature for 45 m, after which they were stained in 1% acetic orcein or in Schiff’s reagent at room temperature for more than 2 hours. The root tips were then mounted in 45% acetic acid, macerated and then squashed by hand under a coverslip. Somatic metaphases were then examined from a minimum of five metaphase preparations from 3–6 different individuals using an Olympus microscope U-TV1-X and a C3030 camera. Suitable preparations were then made permanent by freezing in solid CO2 for removing the cover slip, dehydrating in ethanol and mounting in Canada balsam.

### Stomata measurements

Ploidy level was also indirectly estimated by measurement of stomata size. For the five populations for which chromosome counts were unavailable by lack of adequate material (living plants or achenes), measurements were carried out on five individuals from desiccated leaves of the same individuals used for microsatellite analysis. For the rest of populations, one to three individuals were used. Foliar segments were excised from the middle part of the leaf, softened in NaOH 0.5 M for 24 h, and bleached in a saturated solution of chloral hydrate for 6–12 h. Epidermis fragments were excised from the adaxial side of the distal half of the segments, semipermanently mounted in glycerol sealed with nail varnish, and measured with a micrometric ocular mounted in an Olympus CH-2 optic microscope at 400x. Two measures, long axis (stoma length or SL) and short axis (stoma width or SW), were made on an ideal ellipse adapted to the stoma. Measurement of SL equals the length of the guard cell. For each mounted slide, measurements were made on different zones of the epidermis of the segment, discarding stomata on the margins of the leaf. A total of 30–63 stomata were measured per population. For the five populations for which we lacked chromosome counts, 100–120 stomata were measured.

### DNA isolation and microsatellite loci

Total genomic DNA was extracted from desiccated leaves using the CTAB method of [35], as modified by [36] and [37], and stored at -21°C prior to genotyping. Sixteen SSR markers previously developed for *A*. *millefolium* from Iran [38] were tested for amplification. Seven of these were polymorphic and successfully amplified for yarrow populations from Iberia. All SSR loci were amplified using FAM, NED, PET and VIC fluorescently labeled forward primers as explained by [39]. Different profiles were used for the amplification following the conditions established for each locus in the original publication. Given the importance of clonal propagation in *A*. *millefolium*, plants were collected at least 1 meter apart to avoid duplication. For this reason, we obtained data for 12–16 random individuals per site, accounting for a total of 375 individuals belonging to 26 populations (Fig 1) and representing the entire distribution area of the species and the four cytotypes known to occur within the Iberian Peninsula. Vouchers were deposited at the herbarium of the Botanic Institute of Barcelona (BC). Genotyping was performed by means of an ABI 3730xl DNA Analyzer (Applied Biosystems, Foster City, CA, USA) and LIZ600 size standard at the Interdisciplinary Center for Biotechnology Research (ICBR) facility at the University of Florida. Fragment analysis was performed using GENEMARKER 1.5 software (SoftGenetics, LLC, State College, PA) and data was scored manually.

### Statistical analysis

As mentioned in the introduction, polyploidy frustrates the statistical treatment of microsatellite data and prevents the performance of many analyses and calculations, especially when individuals with different ploidy levels co-exist in the same matrix, as in the present work [22–24,31–34]. We intended to use peak heights to determine the multilocus genotypes of each individual [40], but we were not able to do so for individuals other than for homozygous and fully heterozygous individuals. For this reason, and since there were four different ploidy levels (2*x*, 4*x*, 6*x* and 8*x*), we followed the approach of [32] and recorded the banding patterns observed at each locus as a binary presence/absence matrix: alleles were coded as a separate “locus” into 0 (allele absent) vs. 1 (allele present); the so called ‘allele phenotypes’. Therefore, despite using co-dominant markers for amplification and genotyping, data were scored as dominant loci in order to render them suitable for conventional population genetics analysis software. Although this method may imply some bias, we considered it the best way to treat individuals having different ploidy levels in the same matrix when dosage effect was not evident. However, measures of genetic diversity, such as total number of alleles and number of private alleles, would not be biased [32].

All the genetic diversity parameters were computed using GenAlEx 6.5 [41,42]. At population level, the total number of alleles (*k*), the mean number of alleles (*Na*), the number of effective alleles (*Ne*), the number of private alleles (*PA*) and the unbiased diversity (*uh*) were calculated. These parameters were also computed at ploidy level. The genetic relationship between pairwise populations and ploidy levels was estimated by means of Nei’s unbiased genetic distance (*D*) and Φ_PT_, a measure of population genetic differentiation analogous to F_ST_ (GenAlEx documentation). The significance level of Φ_PT_ was computed through 99 permutations.

Four different approaches were used in order to gain insights into the patterns of spatial genetic structure of *A*. *millefolium* in Iberia. Firstly, the Bayesian method implemented in the software Structure 2.2 [43] was used to infer the number of genetic clusters present in the data set. The iterations were conducted using the admixture model with correlated allele frequencies, since we assumed that a certain amount of gene flow occurred between populations. The analyses were executed with both prior information on population structure and with no a-priori structure to evaluate the consistency of the groups obtained. We carried out an exploratory run using the number of clusters (K) ranging from 1 to 28 (the total number of populations of the study plus two), using only 10^4^ Markov MonteCarlo (MCMC) replications. This analysis did not yield any structure in the data. We carried out a second analysis to see whether we could improve the results, restricting K to a range varying from K = 1 to K = 15. Each K was estimated 10 times, the length of burn-in period comprising 10^5^ iterations and 10^6^ MCMC replications. The optimal number of clusters was determined using the ΔK statistical approach of [44]. Structure assumes Hardy-Weinberg equilibrium within populations and linkage equilibrium between loci within populations [43] and, given the polyploid nature of our data, these tests could not be computed. In order to confirm the results yielded by Structure, we also applied a non-model-based approach: nonhierarchical K-means clustering [45], as implemented in [46]. This approach, free from a priori population genetic assumptions, assigns individuals to a defined number of genetic groups (K) in order to maximize the intergroup variance (measured here as the inertia [47]). The analysis was performed using R (package "stats" [48]), based on the script of [46]. We performed 100000 independent runs for each assumed value of K (ranging from 1 to 15). We computed I(K) and sd(K), the mean and standard deviation of inter-group inertia for each K value and then I1(K) and I2(K), the first and second order derivatives of I(K). We computed ∆K as L2(K) divided by sd(K) and used it as a criterion for selecting the most likely number of groups in our clustering analyses.

The remaining three methods were performed using GenAlEx 6.5. software. An analysis of molecular variance (AMOVA) was computed with respect to three different factors: 1) between and within populations; 2) between and within ploidy levels; and 3) between and within soil type (calcareous vs. siliceous). In addition, a Principal Co-ordinate Analysis (PCoA) was performed based on the pair-wise genetic distances of individuals and populations. Finally, isolation-by-distance between populations was investigated by computing the correlation between the matrix of pair-wise population genetic distance (Φ_PT_) and the matrix of geographical distances, by applying the Mantel test (1000 permutations).

## Results

**Fig 2:**
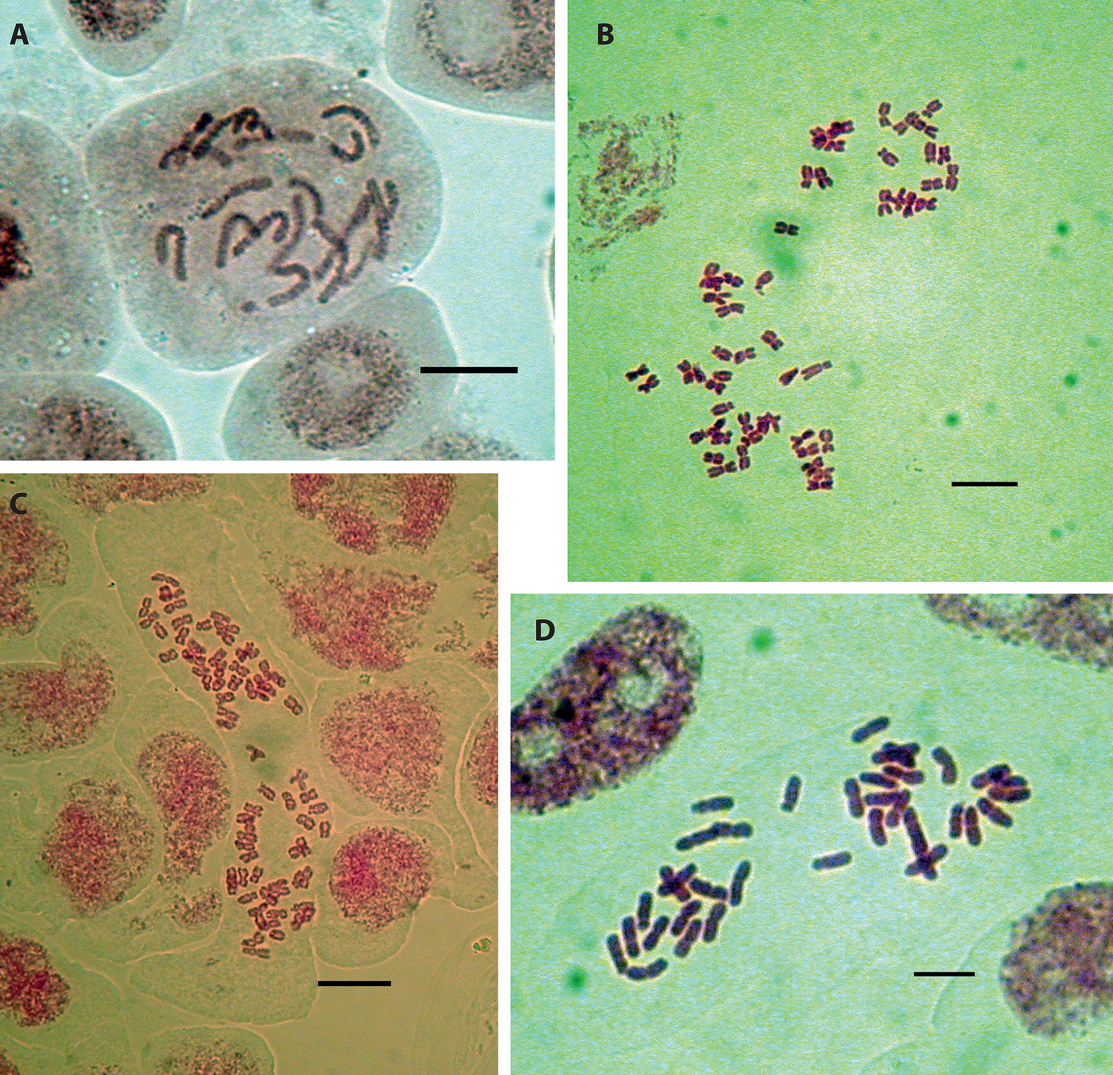
Selected metaphases of the four ploidy levels. A) Diploid plate with 2*n* = 18 (pop. Gi1); B) Hexaploid plate with 2*n* = 54 (pop. Sa2); C) Octoploid plate with 2*n* = 72 (pop. Cs1); D) Tetraploid plate with 2*n* = 36 (pop. Pa1). Scale bar = 10 μm

Chromosome numbers are summarized in Table 1, and partly illustrated in Fig 2. Stomata sizes and statistics of the measurements are shown in supplementary information S1, S2 and S3 Tables, and S1 Fig. The seven SSR primer pairs used yielded 107 alleles. The overall unbiased diversity (*uh*) for *A*. *millefolium* was 0.077. At population level (Table 2), the total number of alleles (*k*) ranged from 16 (Hu1) to 35 (B1, Le1, Lu1 and Na2), the mean number of alleles (*Na*) ranged from 0.224 (Hu1) to 0.645 (B1) and the number of effective alleles (*Ne*) ranged from 1.032 (Hu1) to 1.169 (B1). Forty-five exclusive alleles were detected ranging from one (B2, Cs1, Hu2, Le1, Pa1, S2 and Te1) to six (HU3), all of them occurring at low frequencies (mean frequency 0.097). Only five of the populations surveyed lacked private alleles (Hu1, Le2, Lo1, Na1 and Sa1). The lowest value for unbiased diversity was found in population Hu1 (*uh* = 0.022), whereas the highest value was found in population B1 (*uh* = 0.112). Nei’s genetic distance (*D*) between pairs of populations ranged from 0.002 between L1 and Sa1 to 0.049 between Gi1 and Lo1 (see Table 3 for details). Most values of pair-wise population genetic differentiation Φ_PT_ were significant (P < 0.05) and ranged from 0.044 between B1 and S2 to 0.334 between Lu1 and S1 (see Table 3 for details).

**Table 2:** Main parameters of genetic diversity for the 26 populations surveyed and the four ploidy levels of Spanish *A*. *millefolium*. N, number of individuals; *k*, total number of alleles; *Na*, mean number of alleles; *Ne*, number of effective alleles; *PA*, number of private alleles; *uh*, unbiased diversity. For abbreviations of populations, see Table 1.

Population	N	*k*	*Na*	*Ne*	*PA*	*uh*
Av1	15	34	0.636	1.158	2	0.105
B1	12	35	0.645	1.169	2	0.112
B2	15	24	0.402	1.108	1	0.066
Cs1	15	27	0.458	1.124	1	0.077
Gi1	11	22	0.383	1.109	2	0.071
Gu1	15	32	0.589	1.119	3	0.084
Hu1	14	16	0.224	1.032	0	0.022
Hu2	15	30	0.533	1.108	1	0.074
Hu3	15	34	0.607	1.095	6	0.070
Hu4	15	32	0.589	1.144	2	0.094
L1	12	24	0.421	1.100	2	0.068
Le1	14	35	0.598	1.095	1	0.070
Le2	15	29	0.505	1.125	0	0.082
Lo1	15	20	0.327	1.081	0	0.051
Lu1	15	35	0.617	1.123	2	0.083
M1	15	30	0.542	1.127	3	0.084
Na1	16	22	0.383	1.082	0	0.054
Na2	15	35	0.626	1.141	2	0.094
Pa1	14	29	0.505	1.141	1	0.088
S1	15	31	0.533	1.098	2	0.071
S2	15	30	0.523	1.125	1	0.080
Sa1	13	28	0.505	1.138	0	0.091
Sa2	15	33	0.607	1.162	4	0.104
T1	15	26	0.458	1.117	3	0.075
Te1	14	24	0.411	1.102	1	0.064
Vi1	15	33	0.570	1.094	3	0.067
**Total**	**375**	**107**	**0.508**	**1.116**	**45**	**0.077**
2*x*	11	22	0.383	1.109	2	0.071
4*x*	14	29	0.505	1.141	1	0.088
6*x*	102	76	0.567	1.133	25	0.088
8*x*	248	77	0.485	1.106	26	0.071

**Table 3:** Pair-wise population matrix. Nei’s unbiased genetic distance (D) is shown below diagonal and values for population genetic differentiation (Φ_PT_) are shown above diagonal. For abbreviations of populations, see Table 1. *P<0.05.

	Av1	B1	B2	Cs1	Gi1	Gu1	Hu1	Hu2	Hu3	Hu4	L1	Le1	Le2	Lo1	Lu1	M1	Na1	Na2	Pa1	S1	S2	Sa1	Sa2	T1	Te1	Vi1
Av1	0.000	0.131*	0.062*	0.169*	0.105*	0.187*	0.168*	0.089*	0.189*	0.106*	0.025	0.140*	0.091*	0.191*	0.216*	0.199*	0.125*	0.113*	0.032	0.101*	0.164*	0.000	0.106*	0.056	0.158*	0.106*
B1	0.020	0.000	0.088*	0.120*	0.101*	0.062*	0.136*	0.071*	0.069*	0.075*	0.123*	0.079*	0.147*	0.142*	0.103*	0.099*	0.122*	0.046*	0.165*	0.250*	0.044*	0.151*	0.110*	0.059*	0.068*	0.088*
B2	0.021	0.017	0.000	0.111*	0.104*	0.095*	0.118*	0.012	0.121*	0.015	0.050	0.074*	0.076*	0.116*	0.133*	0.147*	0.125*	0.037	0.086*	0.160*	0.069*	0.039	0.119*	0.084*	0.054	0.022
Cs1	0.027	0.018	0.024	0.000	0.180*	0.104*	0.209*	0.095*	0.130*	0.149*	0.194*	0.148*	0.205*	0.187*	0.120*	0.156*	0.174*	0.076*	0.214*	0.295*	0.101*	0.183*	0.158*	0.128*	0.111*	0.094*
Gi1	0.038	0.021	0.040	0.045	0.000	0.141*	0.175*	0.082*	0.175*	0.140*	0.069	0.180*	0.124*	0.237*	0.221*	0.155*	0.148*	0.099*	0.105*	0.165*	0.146*	0.107*	0.112*	0.087*	0.148*	0.133*
Gu1	0.031	0.009	0.014	0.019	0.032	0.000	0.118*	0.061*	0.030	0.132*	0.181*	0.067*	0.161*	0.137*	0.020	0.073*	0.189*	0.050*	0.200*	0.267*	0.046*	0.180*	0.123*	0.082*	0.073*	0.079*
Hu1	0.026	0.023	0.023	0.036	0.031	0.020	0.000	0.067*	0.101*	0.190*	0.173*	0.182*	0.254*	0.194*	0.135*	0.226*	0.151*	0.116*	0.220*	0.316*	0.112*	0.164*	0.145*	0.100*	0.154*	0.047*
Hu2	0.024	0.017	0.015	0.020	0.034	0.014	0.013	0.000	0.036	0.044	0.067	0.094*	0.083*	0.097*	0.097*	0.110*	0.075*	0.033	0.119*	0.177*	0.058*	0.074*	0.109*	0.065*	0.025	0.000*
Hu3	0.027	0.012	0.018	0.016	0.035	0.007	0.015	0.004	0.000	0.146*	0.168*	0.083*	0.175*	0.103*	0.053*	0.150*	0.112*	0.057*	0.214*	0.274*	0.066*	0.196*	0.141*	0.074*	0.067*	0.065*
Hu4	0.028	0.006	0.015	0.022	0.035	0.011	0.035	0.020	0.016	0.000	0.081	0.080*	0.075*	0.193*	0.165*	0.150*	0.140*	0.043	0.116*	0.189*	0.097*	0.082*	0.179*	0.085*	0.042	0.089*
L1	0.015	0.007	0.012	0.015	0.018	0.008	0.012	0.012	0.007	0.018	0.000	0.090*	0.011	0.194*	0.215*	0.179*	0.100*	0.080*	0.026	0.032	0.164*	0.004	0.170*	0.054	0.154*	0.105*
Le1	0.030	0.015	0.013	0.020	0.046	0.005	0.027	0.020	0.011	0.013	0.011	0.000	0.075*	0.174*	0.077*	0.103*	0.167*	0.030	0.109*	0.174*	0.076*	0.136*	0.155*	0.079*	0.089*	0.109*
Le2	0.036	0.011	0.020	0.023	0.042	0.009	0.035	0.020	0.014	0.011	0.016	0.005	0.000	0.226*	0.215*	0.142*	0.159*	0.093*	0.046	0.028	0.179*	0.051	0.235*	0.095*	0.149*	0.159*
Lo1	0.032	0.022	0.017	0.025	0.049	0.019	0.026	0.015	0.013	0.030	0.018	0.026	0.031	0.000	0.163*	0.225*	0.216*	0.140*	0.257*	0.323*	0.169*	0.208*	0.184*	0.156*	0.200*	0.121+
Lu1	0.028	0.015	0.017	0.019	0.039	0.003	0.018	0.013	0.007	0.015	0.007	0.006	0.011	0.019	0.000	0.147*	0.211*	0.066*	0.256*	0.334*	0.056*	0.217*	0.159*	0.118*	0.112*	0.094*
M1	0.047	0.020	0.037	0.026	0.040	0.016	0.044	0.030	0.029	0.028	0.024	0.020	0.020	0.039	0.023	0.000	0.220*	0.091*	0.178*	0.235*	0.132*	0.182*	0.178*	0.123*	0.145*	0.168*
Na1	0.024	0.019	0.023	0.022	0.034	0.027	0.019	0.011	0.013	0.022	0.013	0.028	0.026	0.031	0.024	0.041	0.000	0.091*	0.143*	0.237*	0.159*	0.163*	0.184*	0.069*	0.085*	0.097*
Na2	0.021	0.012	0.017	0.010	0.031	0.011	0.025	0.018	0.012	0.014	0.006	0.007	0.010	0.026	0.009	0.024	0.015	0.000	0.123*	0.207*	0.011	0.105*	0.097*	0.058*	0.017	0.021*
Pa1	0.019	0.019	0.016	0.023	0.030	0.020	0.026	0.020	0.021	0.020	0.013	0.011	0.016	0.037	0.022	0.027	0.020	0.012	0.000	0.019	0.206*	0.020	0.190*	0.076	0.176*	0.168*
S1	0.033	0.016	0.022	0.021	0.028	0.008	0.025	0.017	0.008	0.026	0.009	0.007	0.011	0.029	0.014	0.016	0.025	0.012	0.010	0.000	0.298*	0.034	0.298*	0.132*	0.277*	0.258*
S2	0.019	0.007	0.013	0.016	0.029	0.009	0.021	0.017	0.011	0.015	0.006	0.007	0.013	0.025	0.007	0.027	0.019	0.008	0.015	0.012	0.000	0.175*	0.092*	0.111*	0.004	0.032*
Sa1	0.009	0.009	0.017	0.018	0.025	0.009	0.010	0.013	0.010	0.024	0.002	0.012	0.016	0.019	0.008	0.024	0.020	0.007	0.011	0.009	0.010	0.000	0.175*	0.070	0.175*	0.114*
Sa2	0.006	0.022	0.023	0.033	0.022	0.025	0.026	0.024	0.025	0.031	0.013	0.027	0.034	0.031	0.028	0.037	0.029	0.022	0.019	0.024	0.018	0.011	0.000	0.132*	0.138*	0.089*
T1	0.018	0.009	0.022	0.014	0.023	0.008	0.018	0.014	0.008	0.014	0.008	0.018	0.018	0.026	0.011	0.025	0.015	0.011	0.022	0.015	0.012	0.010	0.023	0.000	0.095*	0.099*
Te1	0.026	0.014	0.014	0.018	0.035	0.015	0.030	0.012	0.012	0.007	0.014	0.014	0.014	0.033	0.017	0.032	0.009	0.012	0.016	0.019	0.007	0.025	0.028	0.014	0.000	0.038*
Vi1	0.013	0.019	0.012	0.021	0.037	0.019	0.010	0.009	0.010	0.026	0.005	0.018	0.020	0.018	0.015	0.042	0.010	0.012	0.015	0.018	0.014	0.007	0.020	0.016	0.016	0.000

Regarding ploidy level (Table 2), the total number of alleles (*k*) ranged from 22 (2*x*) to 77 (8*x*), the mean number of alleles (*Na*) ranged from 0.383 (2*x*) to 0.567 (6*x*) and the number of effective alleles (*Ne*) ranged from 1.106 (8*x*) to 1.141 (4*x*). Fifty-five exclusive alleles were detected ranging from one (4*x*) to 28 (8*x*). The lowest value for unbiased diversity belonged to 2*x* and 8*x* (*uh* = 0.071) cytotypes, whereas the highest value belonged to 4*x* and 6*x* (*uh* = 0.088) cytotypes. Nei’s genetic distance (*D*) between ploidy levels ranged from 0.002 between 6*x* and 8*x* to 0.030 between 2*x* and 4*x* (see Table 4 for details). All values of genetic differentiation Φ_PT between_ ploidy levels were significant (P < 0.05) and ranged from 0.005 between 6*x* and 8*x* to 0.105 between 2*x* and 4*x* cytotypes (see Table 4 for details).

**Table 4:** Pair-wise ploidy matrix. Nei’s unbiased genetic distance (D) is shown below diagonal and values for genetic differentiation (Φ_PT_) are shown above diagonal. *P<0.05.

	2*x*	4*x*	6*x*	8*x*
**2*x***	0.000	0.105*	0.065*	0.086*
**4*x***	0.030	0.000	0.080*	0.093*
**6*x***	0.024	0.010	0.000	0.005*
**8*x***	0.026	0.011	0.002	0.000

**Fig 3:**
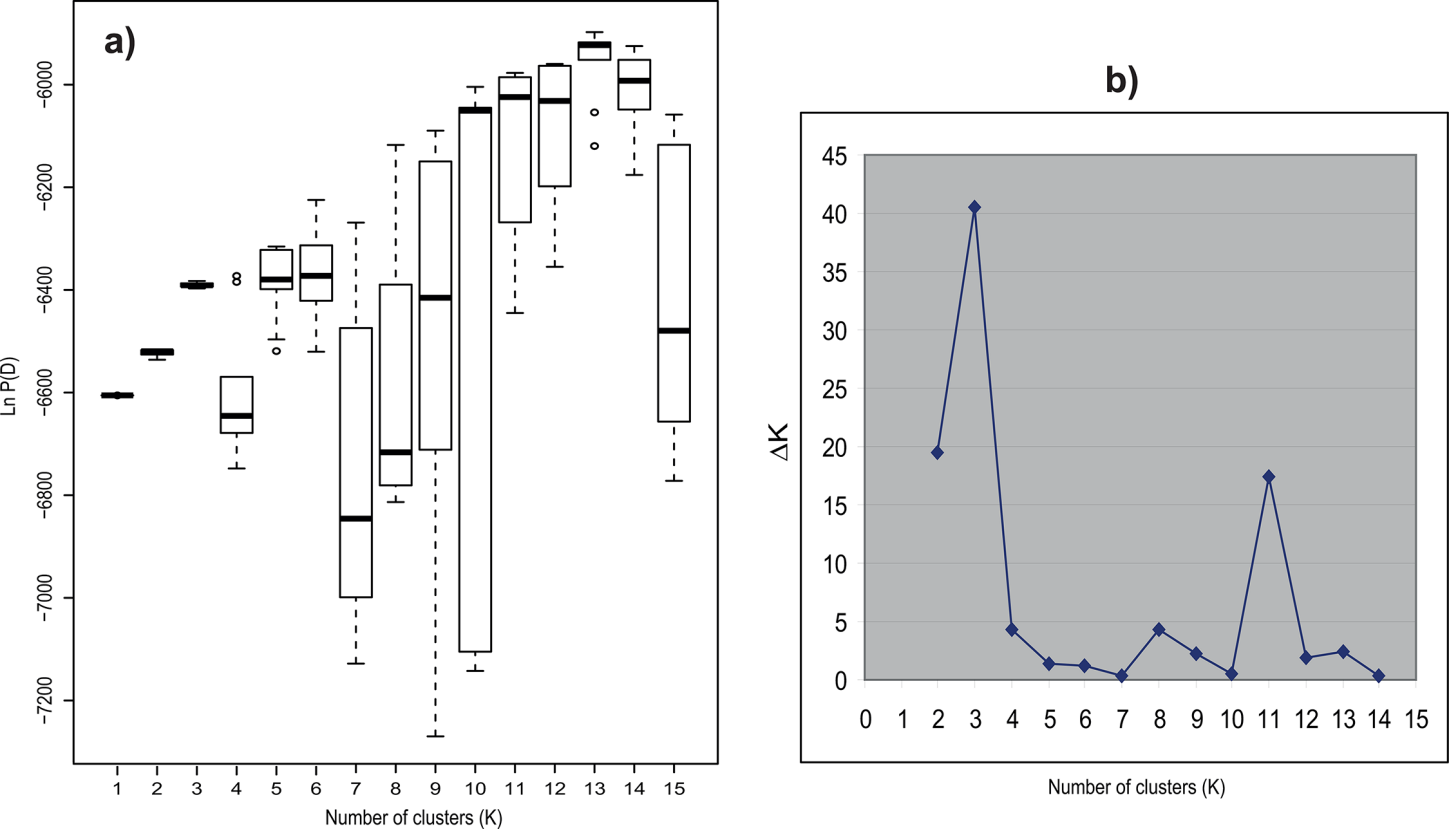
a) Ln P(D), mean (±standard deviation) of log-likelihood values for each value of K = 1–15 (10 independent runs per K). **b) Mean absolute difference of the second order rate of change with respect to K (ΔK, following [44]).** Most supported K value: K = 3.

**Fig 4:**
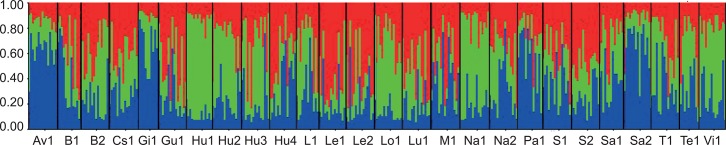
Percentage assignment of each individual (represented by vertical bars) to each of the three genetic clusters (represented by different colours) inferred by the program Structure [43]. See Table 1 for population codes.

**Fig 5:**
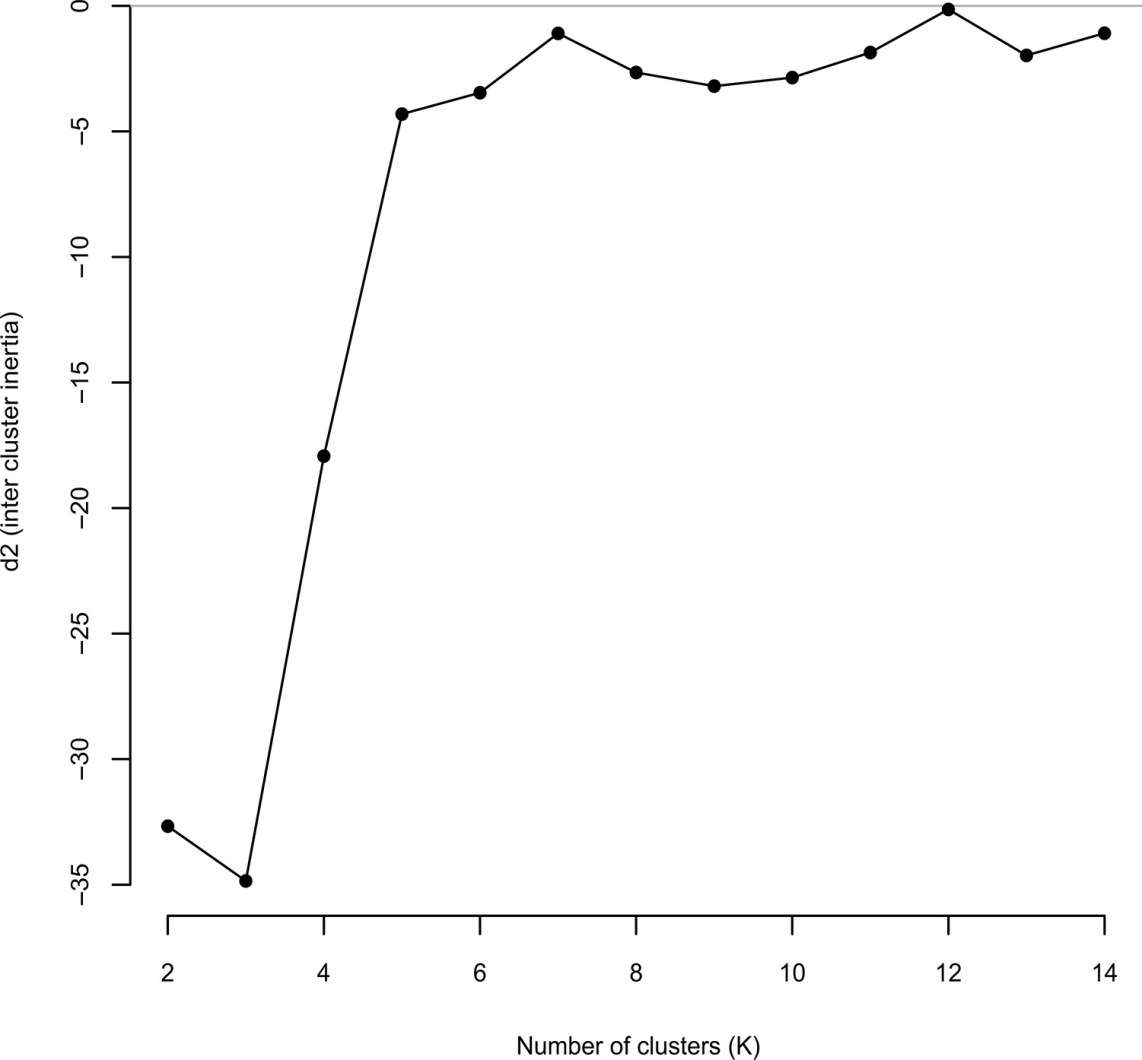
Screening for the most likely number of groups (K) with non-hierarchical K-means clustering [45], performed with 100000 independent runs for each value of K. The runs maximising ΔK values were initially considered as optimal (K = 7 and K = 12), and because K = 12 added little gain to ΔK, we kept K = 7 as the optimal final value.

**Fig 6:**
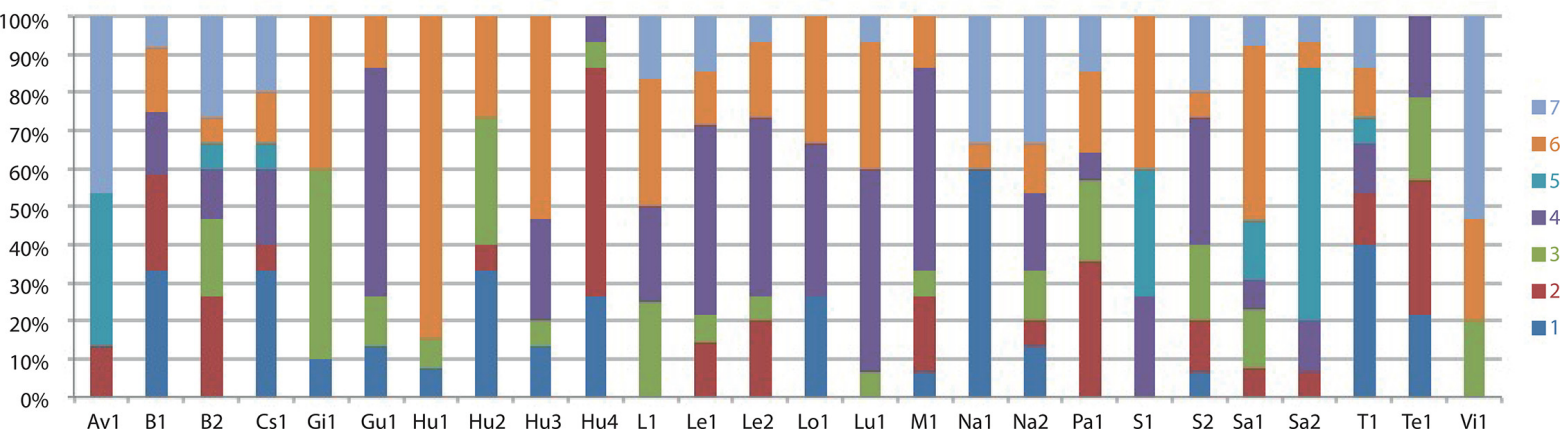
Percentage of individuals that belong to each of the seven genetic clusters (represented by different colours) inferred by the nonhierarchical K-means clustering analysis [46]. Each vertical column corresponds to a population. See Table 1 for population codes.

**Fig 7:**
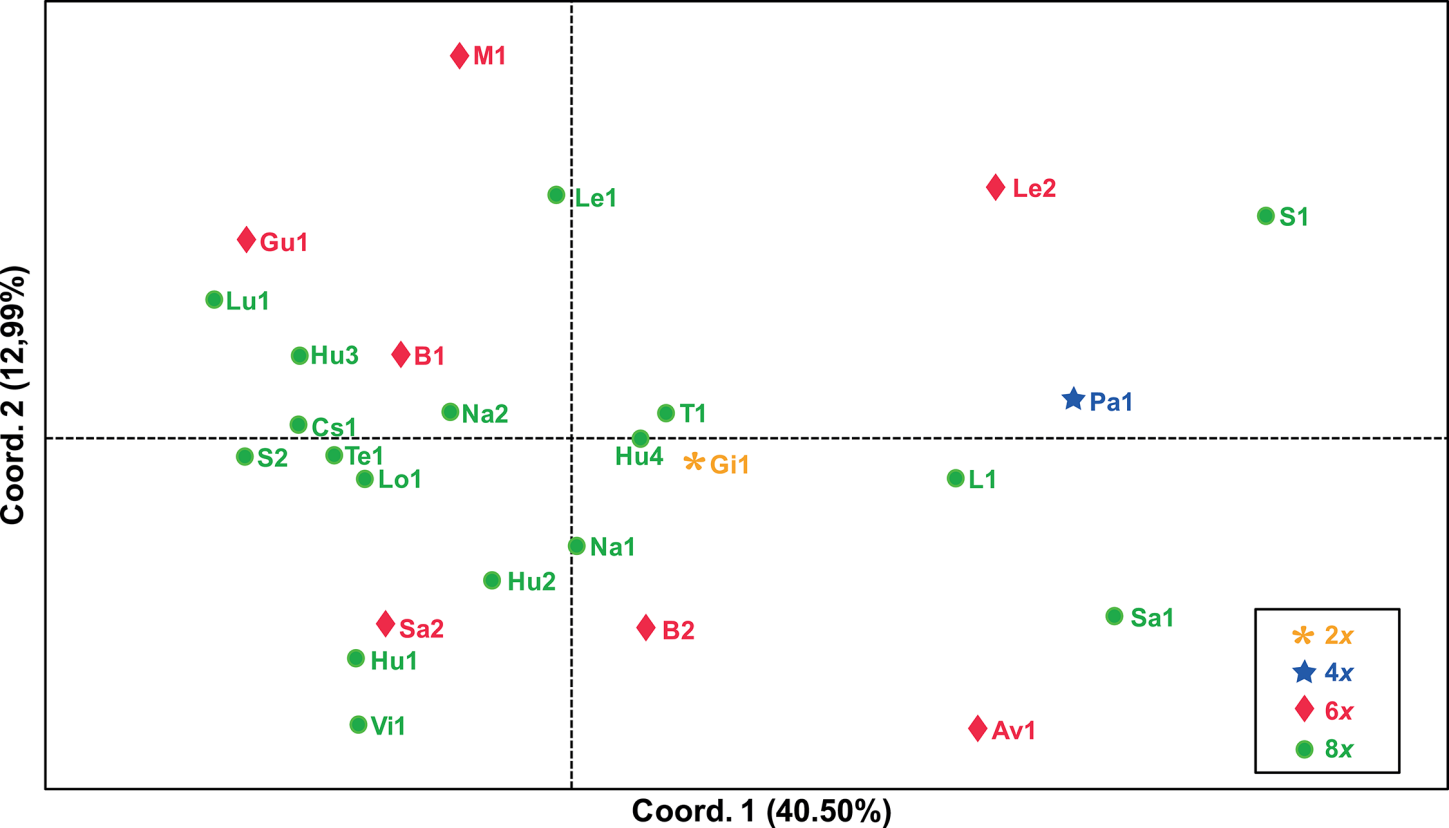
Principal component analysis performed from pairwise Nei’s genetic distances between populations. Orange asterisk: 2*x*; blue star: 4*x*; pink diamond: 6*x*; green dot: 8*x*. See Table 1 for population codes.

**Fig 8:**
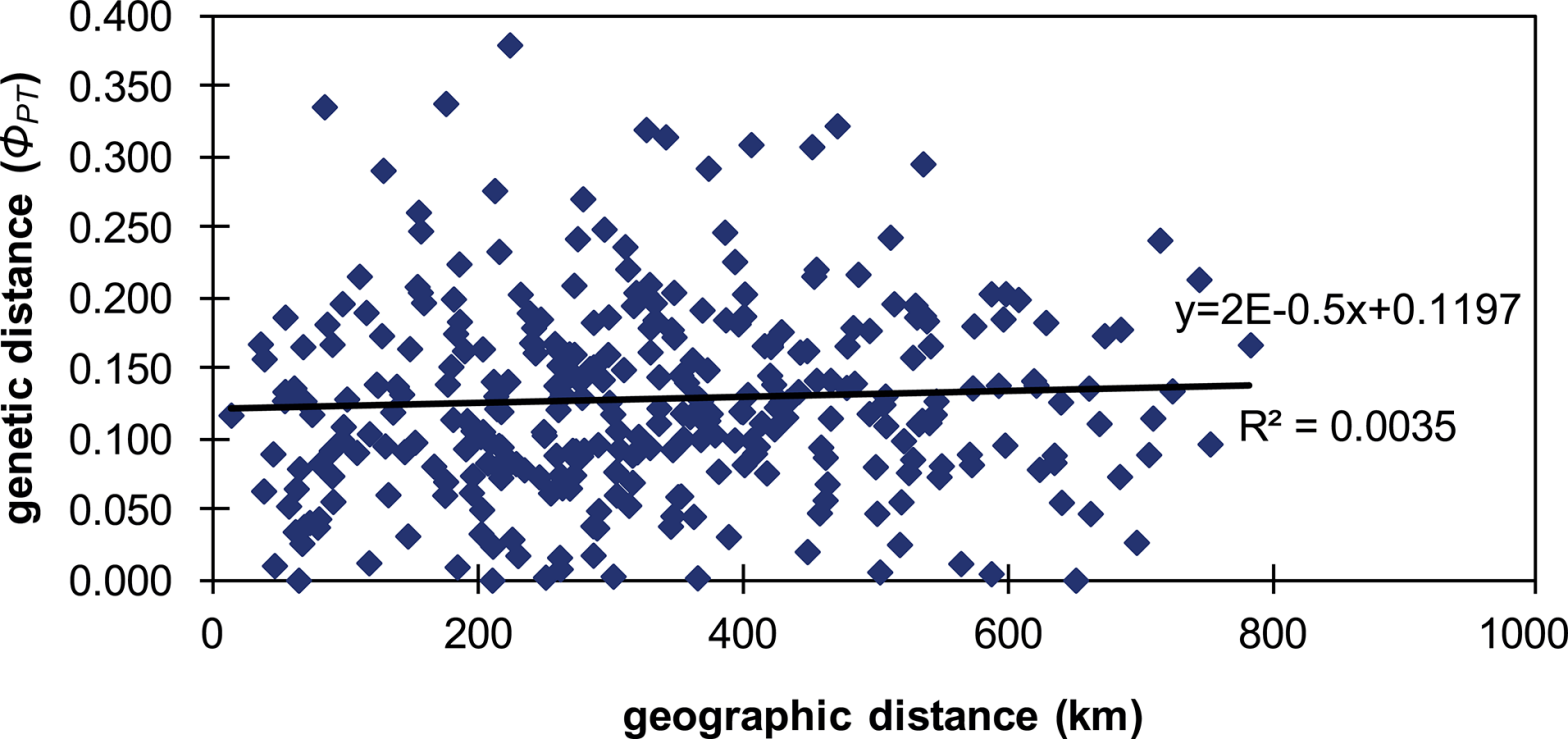
Mantel test correlating geographic distance and genetic distance. Results of the IBD test searching for a correlation between geographic distance and genetic distance.

The results of the second Structure simulation were consistent between runs and, according to [44], K = 3 was the most likely number of clusters for our data (Fig 3). Despite this, no population clusters were generated in any of the runs performed; instead each population was subdivided into three groups (Fig 4). The same pattern was recovered for all the Ks tested. In contrast, the nonhierarchical K-means clustering analysis yielded K = 7 as the most likely according to [44] (Fig 5), but again the resulting groups yielded no population clusters (Fig 6). This absence of population structuring was also found in the other Ks assessed. When AMOVA was performed at population level, the majority of variations (87%) was significantly partitioned within populations, while 13% of the variation was detected between populations (Φ_PT_ = 0.126; *P* < 0.005). Similarly, when the partition was based on the ploidy level, most of the variation was again detected within groups (97%), while only 3% of the variation was detected between ploidy levels (Φ_PT_ = 0.026; *P* < 0.005). Finally, the same pattern was detected when considering soil type, since 99% of the variation was detected within groups, while only 1% of the variation was detected between the two soil types (Φ_PT_ = 0.012; *P* < 0.05). With regard to the PCoA analysis, the first three components explain 43.27% and 64.55% of total variance of data when considering individuals and populations, respectively. At individual level, in the scatter-plot for the two first components (35.70% of the variance), individuals are distributed across the graph without any grouping pattern, regardless of the population to which they belong (S2 Fig). Similarly, a lack of clustering pattern is again recovered when considering populations (Fig 7) in the scatter-plot for the two first components (53.49% of the variance). No clusters of individuals or populations were detected based either on their geographic distribution, or their chromosome number (Fig 7). According to the Mantel test (Fig 8), no correlation was detected between genetic and geographic distance, and therefore, the hypothesis of isolation by distance (IBD) was rejected (Rxy = 0.059; P = 0.198).

## Discussion

Our results for Bayesian clustering (Figs 4 and 6), nonhierarchical clustering, AMOVA (Table 4) and PcoA (Fig 7 and S2 Fig), as well as the low values of pair-wise population differentiation, pair-wise genetic distance and the absence of IBD (Fig 8) all suggest the existence of a substantial panmictic yarrow population in the Iberian Peninsula: although this species presents displays clonal reproduction, outbreeding predominates [14], and this is consistent with panmixia. The weak geographical genetic structure recovered is linked to the high levels of gene flow between populations [46] and to the fact that most of the genetic variability is found within populations. These results are also consistent with the assumption that a recent colonization event and rapid expansion of the species occurred throughout the Iberian Peninsula involving especially octoploid and hexaploid cytotypes. Population structure usually develops by means of population genetic differentiation, either by adapting to local conditions or via random genetic drift. However, this process is slow and there has been too little time since colonization for this to be reflected in neutral markers such as microsatellites [49]. With reference to local adaptation, *A*. *millefolium* in Iberia grows only in ruderal and nitrified environments, and this ecological uniformity may, in turn, have introduced similar adaptive pressures to the entire colonized area and may, in part, have been responsible for the genetic uniformity of this species. Moreover, a study investigating the colonization of North America [50], where yarrow displays maximum ecological diversity within its extensive distribution range, recovered low levels of genetic diversity and negligible genetic structure (associated with varietal identity, geographical distribution or ploidy level), as well as maximum variation in terms of intra-population differences. To summarize, virtually the same genetic pattern that we recovered in the Iberian Peninsula was found for North America [50]. Given that the great taxonomic diversity present in North America is interpreted to be mainly due to ecological adaptation to habitat differences (such as in the case of ecological specialists like *A*. *millefolium* var. *gigantea* or *A*. *millefolium* var. *puberula*) and phenological differences (for example, between *A*. *millefolium* var. *arenicola* and *A*. *millefolium* var. *littoralis*), the few taxa comprising Iberian *A*. *millefolium* agg. may be due to their uniform ecological preferences (i.e., ruderal and disturbed habitats).

Similarly, the low levels of genetic variability recovered suggest that bottlenecks and/or founder events may have been involved in this process [51,52]. When a population passes through a bottleneck, its genetic diversity is expected to diminish to a greater or lesser degree depending on both the size of the founding population and the rate of population growth. However, when population size increases, genetic diversity may increase by the occurrence of new mutations [51]. In our opinion, the presumed genetic influence of population size and growth following introduction of *A*. *millefolium* to the Iberian Peninsula has not had sufficient time to produce new variations and to develop a clear genetic structure [49]. Furthermore, this process may have been counter-balanced by the influence of clonal reproduction, which may also have played an important role in maintaining genetic impoverishment [53]. Previous studies of *A*. *millefolium* agg. phylogeny based on both nuclear and plastid genomes found low levels of genetic variation and limited resolution [12]. According to [54], the genetic consequences of the colonization of a new area are influenced not only by the number of introductions, but also by the number of propagules introduced at each introduction event. Low levels of genetic variation are commonly explained by few introductions and small founding population sizes [54,55]. Conversely, a pattern of great genetic diversity within populations is explained by the occurrence of multiple introductions from different genetic lineages present in the indigenous area [54,56–60]. Our results suggest that the number of both colonization events and propagules may have been low during the Iberian colonization by *A*. *millefolium*, and thus, the Iberian Peninsula should be considered both in evolutionary and geographical terms, a dead-end for this species. However, given its therapeutic properties and ornamental value, several independent, human-mediated introductions (intentionally or accidentally by livestock) cannot be ruled out, and this may explain both the low levels of genetic diversity and the presence of exclusive alleles found in most of the populations studied.

Another factor that may affect genetic variability is invasiveness. For example, *Rubus alceifolius* displays least genetic diversity in areas where it has become a serious weed [55], and it has been suggested that a genotype well-adapted to a specific biotope may spread rapidly through an area by means of asexual reproduction. *Achillea millefolium* is considered such a weed and meets the parameters attributed to invasive plants [61], namely, great asexual reproductive potential and high ploidy levels. According to our data, the behavior of *A*. *millefolium* during the colonization of the Iberian Peninsula may be considered as invasive.

With reference to the correlation between ploidy level and genetic diversity in *A*. *millefolium*, it is expected to be greater for polyploids. Polyploidy allows more recombination events to occur during meiosis and therefore results in increased diversity of the genome, thus allowing adaptive plasticity [24,62] and reducing the cost of inbreeding [55]. According to our results, yarrow from the Iberian Peninsula does not display this pattern. Consistently, [3] found that throughout Europe, the number of AFLP bands per individual were not significantly greater in polyploid genomes than in those of diploids and suggested that this may indicate genome down-sizing or sequence suppression during polyploidization. These authors also discovered that the number of polymorphic bands was significantly greater for polyploid populations compared to diploid populations, suggesting a greater degree of genetic polymorphism in polyploids. Indeed, the low levels of genetic differentiation between ploidy levels recovered in our work also suggest an absence of barriers between them, even between diploids and the remaining cytotypes. Negligible genetic differentiation was also observed to exist between 4*x* and 6*x* ecotypes of *A*. *millefolium* agg. in America [50] and this [3] highlighted the existence of hybrid contacts and several independent lines of auto- and allopolyploidy both within the same and between different ploidy levels of *A*. *millefolium* agg. This may have masked the separation between sympatric populations and highlights the lack of barriers between polyploids, even those of different species. Consequently, previously published karyological data [63–65], prove that karyotypes of *A*. *millefolium* agg. are quite similar, show limited structural differentiation and lack obvious obstacles to hybridization.

## Conclusions

All of our results indicate a very recent colonization of the Iberian Peninsula by the *Achillea millefolium* aggregate, and this involved a limited number of individuals. The species behaves in Iberia as an invasive plant colonizing ruderal and disturbed habitats. The low genetic variability of populations and lack of differentiation between the different ploidy levels all indicate that the Iberian Peninsula constitutes a geographical cul-de-sac for the expansion of the aggregate and a genetic and evolutionary dead-end for the Iberian populations.

## Supporting Information

S1 Dataset Binary-coded matrix of the microsatellite results for the studied populations. 0 = absence; 1 = presence. See Table 1 for population codes.
(TXT)

S1 Fig Box-plots of the measurements of the stomata. SL = Stomata length; SW = Stomata width.
(EPS)

S2 Fig Principal component analysis performed from pairwise Nei’s genetic distances between individuals. See Table 1 for population codes.
(EPS)

S1 Table Dimensions of stomata for individual populations of known ploidy (by count). See Table 1 for population codes.
(DOCX)

S2 Table Dimensions of stomata for ploidy level. See Table 1 for population codes.
(DOCX)

S3 Table Dimensions of stomata and estimated ploidy level for populations withouth counts. See Table 1 for population codes.
(DOCX)
