# Supplementary material for: The Polyploid Series of the Achillea millefolium Aggregate in the Iberian Peninsula Investigated Using Microsatellites
Source: PLoS One. 2015 Jun 19;10(6):e0129861. doi: 10.1371/journal.pone.0129861 (PMC4474640; doi:10.1371/journal.pone.0129861)
Supplement: S1 Table — See Table 1 for population codes. (DOCX) [file pone.0129861.s004.docx]

S1 Table – Dimensions of stomata for individual populations of known ploidy (by count). See Table 1 for codes). SL, stomatal length; SW, stomatal width; m, number of measures; , mean (μm); std, standard deviation; range, variation range (95% of values).

| **Code** | **Ploidy** | **m** | **SL**  **** | **std** | **SW**  **** | **std** |
| --- | --- | --- | --- | --- | --- | --- |
| Gi1 | 2x | 30 | 21.52 | 1.99 | 15.63 | 1.75 |
| Pa1 | 4x | 100 | 32.39 | 2.70 | 23.90 | 3.31 |
| Av1 | 6x | 30 | 28.92 | 1.99 | 22.58 | 1.75 |
| B2 | 6x | 62 | 31.97 | 1.96 | 24.10 | 1.58 |
| Gu1 | 6x | 63 | 31.94 | 1.82 | 24.17 | 1.81 |
| M1 | 6x | 30 | 32.17 | 2.34 | 23.50 | 1.39 |
| Sa2 | 6x | 50 | 36.20 | 2.05 | 25.23 | 2.20 |
| Cs1 | 8x | 32 | 38.60 | 3.20 | 27.22 | 1.37 |
| Hu1 | 8x | 30 | 39.17 | 3.37 | 27.71 | 2.08 |
| Na1 | 8x | 30 | 42.13 | 2.52 | 29.75 | 1.94 |
| Na2 | 8x | 30 | 44.13 | 2.92 | 27.29 | 2.06 |
| S1 | 8x | 30 | 37.63 | 2.03 | 25.88 | 2.15 |
| S2 | 8x | 30 | 45.75 | 2.84 | 31.17 | 2.80 |
| Sa1 | 8x | 30 | 36.22 | 2.19 | 25.29 | 1.86 |
| Vi1 | 8x | 30 | 43.38 | 3.94 | 32.79 | 3.26 |
