# Supplementary material for: The Polyploid Series of the Achillea millefolium Aggregate in the Iberian Peninsula Investigated Using Microsatellites
Source: PLoS One. 2015 Jun 19;10(6):e0129861. doi: 10.1371/journal.pone.0129861 (PMC4474640; doi:10.1371/journal.pone.0129861)
Supplement: S2 Table — See Table 1 for population codes. (DOCX) [file pone.0129861.s005.docx]

S2 Table – Dimensions of stomata for populations of known ploidy (by count). SL, stomatal length; SW, stomatal width; pop, number of populations; , mean (μm); std, standard deviation; range, variation range (95% of values).

|  |  | N of | SL |  |  |  | SW |  |  |
| --- | --- | --- | --- | --- | --- | --- | --- | --- | --- |
| Ploidy |  | pop. |  | std | range (95%) |  |  | std | range (95%) |
| 2x |  | 1 | 21.52 | — | 19.9 – 23.0 |  | 15.63 | — | 14.9 – 17.0 |
| 4x |  | 1 | 32.39 | — | 27.5 – 37.5 |  | 23.90 | — | 18.8 – 30.0 |
| 6x |  | 5 | 32.24 | 2.59 | 27.5 – 37.7 |  | 23.92 | 0.97 | 20.0 – 28.1 |
| 8x |  | 8 | 40.88 | 3.43 | 33.0 – 50.0 |  | 28.39 | 2.62 | 22.5 – 35.0 |
