# Supplementary material for: The Polyploid Series of the Achillea millefolium Aggregate in the Iberian Peninsula Investigated Using Microsatellites
Source: PLoS One. 2015 Jun 19;10(6):e0129861. doi: 10.1371/journal.pone.0129861 (PMC4474640; doi:10.1371/journal.pone.0129861)
Supplement: S3 Table — See Table 1 for population codes. (DOCX) [file pone.0129861.s006.docx]

S3 Table – Dimensions of stomata and estimated ploidy level for populations withouth counts. SL, stomatal length; SW, stomatal width; n, number of individuals measured; m, number of stomatal measures; , mean (μm); std, standard deviation (among individuals).

|  | **Estimated** |  |  | SL |  | SW |  |
| --- | --- | --- | --- | --- | --- | --- | --- |
| **Code** | **ploidy** | n | m |  | std |  | std |
| B1 | **6x** | 5 | 110 | 35,44 | 2,83 | 26,19 | 1,32 |
| Hu2 | **8x** | 4 | 90 | 39,04 | 2,13 | 28,65 | 1,03 |
| Hu3 | **8x** | 6 | 120 | 38,30 | 2,20 | 28,60 | 1,13 |
| Lo1 | **8x** | 5 | 110 | 38,45 | 1,35 | 25,32 | 0,92 |
| Te1 | **8x** | 5 | 110 | 40,56 | 1,11 | 29,06 | 0,74 |
